# Can Body Mass Index Affect Height Growth at Menarche among Girls Receiving Treatment for Early Puberty? A Retrospective Study in Korean Girls

**DOI:** 10.3390/children9010110

**Published:** 2022-01-14

**Authors:** Sun-Jin Lee, Sun-Young Kim, Minsun Kim

**Affiliations:** 1 Department of Pediatrics, Jeonbuk National University Medical School, Jeonju-si 54907, Korea; 082kiki@hanmail.net; 2 Research Institute of Clinical Medicine, Jeonbuk National University-Biomedical Research Institute, Jeonbuk National University Hospital, Jeonju-si 54907, Korea; kim4889@hanmail.net

**Keywords:** central precocious puberty, gonadotropin-releasing hormone agonist, height growth, menarche, body mass index

## Abstract

Gonadotropin-releasing hormone agonist (GnRHa) therapy is used to control puberty progression and it preserves height potential in patients with idiopathic central precocious puberty (ICPP). This study evaluated the correlation between weight and height gain at menarche following GnRHa treatment among girls with ICPP and relatively central early puberty (EP). We investigated height/weight trends and changes in height from diagnosis to menarche in girls with ICPP and EP treated with GnRHa. The mean difference in height (Δheight) from treatment cessation to menarche was 9.79 ± 3.53 cm. Girls were divided into girls with Δheight ≥ 9.79 cm (Group 1) and girls with Δheight < 9.79 cm (Group 2). Although near adult height was significantly higher in Group 1, the mean body mass index (BMI) and weight were significantly lower at diagnosis, treatment discontinuation, and menarche. The BMI and weight at the three time points were negatively correlated with height. Girls with higher BMI at all three time points had slower growth rates during the study period. Considering that BMI and body weight were closely related to Δheight, proper management of BMI and body weight of girls receiving early puberty treatment might contribute to growth during and after GnRHa treatment.

## 1. Introduction

With time, the annual incidence of central precocious puberty (CPP) has increased, and the age of onset of puberty has lowered worldwide [1]. Precocious puberty is defined as the development of secondary sexual characteristics before the age of eight years in girls and nine years in boys. CPP is caused by a hypothalamic gonadotropin-releasing hormone (GnRH) pulse generator with subsequent pulsatile gonadotropin secretion [2]. This condition limits the final adult height owing to rapid growth velocity and early epiphyseal closure [3]. GnRH agonist (GnRHa) therapy is considered the standard treatment for this condition [4]. CPP treatment aims to delay the early progression of puberty, inhibit epiphyseal growth to prevent shorter adult height, and to allow normal sexual maturation to reduce psychosocial problems [4,5].

Previous studies have assessed the signs of poor growth response despite CPP treatment. Moreover, they showed that factors such as age at CPP onset and treatment initiation, bone age (BA) advancement at the time of initiation and end of therapy, duration of therapy, mid-parental height (MPH), height at the initiation and end of therapy, and predicted adult height (PAH) before and after treatment, affected the final adult height of CPP patients treated with GnRHa [6]. Obesity and overweight status in children have an increasing prevalence and are known risk factors for early CPP [7]. Some studies have reported that obese children have accelerated prepubertal height (Ht) growth and bone age (BA), and a decreased pubertal growth spurt compared to non-obese subjects [7]. Furthermore, many reports have described inconsistent relationships between GnRHa therapy and body mass index (BMI) or weight status, including increase [8,9,10,11,12,13,14], no influence [15,16,17,18,19,20,21], or decrease [22,23,24]. However, these associations remain controversial. The follow-up periods of these studies were limited to undertreatment or for a short treatment after therapy cessation [25,26,27]. However, long-term follow-up studies [28,29] focused on BMI changes and total height gain and did not analyze the relationship between growth trends and BMI from the end of treatment to menarche in children with idiopathic CPP (ICPP) and central early puberty (EP) treated with GnRHa. As a growth spurt occurs before menarche, studies investigating factors influencing height from the cessation of GnRH treatment to menarche can contribute to improve final adult height.

To gain a better understanding of the effects of GnRHa treatment on BMI, we focused on the relationship between the discontinuation of GnRHa treatment and menarche and investigated the BMI from diagnosis to menarche and the height difference from the discontinuation of GnRHa treatment to menarche in girls with ICPP and EP who received GnRHa therapy. The subgroup of patients who achieved a greater height difference during this period was compared to subjects with a lower difference as the control group, and we analyzed whether the growth difference between the cessation of therapy and menarche was related to BMI or other clinical factors. 

## 2. Materials and Methods

### 2.1. Study Participants

A retrospective study was conducted on 88 girls with ICPP (27 subjects) and EP (61 subjects), who were monitored at Jeonbuk National University Children’s Hospital between February 2009 and August 2018. These girls were selected from among 143 girls who were treated with GnRHa therapy and subsequently examined after menarche. In addition, the girls had developed secondary sexual characteristics before the age of 8 years. However, 12 girls who received combination therapy consisting of a growth hormone (GH) and GnRHa, 4 who did not have any record of BA at menarche, 15 who were not followed up after menarche, and 24 who were lost to follow-up during treatment were excluded. 

### 2.2. Diagnosis and Treatment Process

ICPP and EP were diagnosed based on the presence of secondary sexual characteristics [30], including BA greater than the chronological age (CA) [30] and peak luteinizing hormone (LH) levels > 5 IU/L according to a standard intravenous GnRH stimulation test [4], and the absence of central nervous system lesions on brain magnetic resonance imaging [4]. GnRHa was administered intramuscularly every 28 days, as a depot formulation of the GnRH analog leuprorelin, at a dose of 0.2–0.3 mg/kg/dose every 4 weeks [4]. GnRHa treatment was initiated before the age of 8 years (ICPP, range: 6.17–7.92) and between 8 and 9 years (EP, range: 8.00–8.92). Treatment was continued for at least 1.5 years and was completed after 12 years of BA [31,32]. All patients underwent clinical and hormonal assessments every six months until treatment completion. At 3 months of treatment, a GnRH stimulation test was performed to define peak LH levels below 2 IU/L to indicate suppression of the hypothalamic-pituitary-gonadal axis [33]. The patients visited the hospital within 3 months of menarche.

### 2.3. Data Collection and Analysis

Physical examinations were performed from the first hospital visit to after-menarche, and Height, weight, BMI, and MPH were expressed as standard deviation scores (SDS) for CA. The SDS values were converted using the 2017 Korean National Growth Charts for children and adolescents [34]. The height and weight measurements were converted to BMI percentiles according to these charts. BMI was calculated by dividing body weight in kilograms by height in meters squared. Overweight was defined as age-and sex-specific BMI ≥ 85th and <95th percentiles, and obesity was defined as a BMI ≥ 95th percentile. 

CA, height, weight, BMI, BA at diagnosis, treatment discontinuation, and menarche were assessed. MPH was evaluated during the first visit by two pediatric endocrinologists. BA was assessed using radiographic images of the left hand using the Greulich–Pyle method. MPH was defined as the average of the parental height minus 6.5 cm. The PAH was calculated using the Bayley–Pinneau method. The hypothalamic–pituitary–ovarian axis was evaluated by measuring basal and stimulated LH and follicle-stimulating hormone (FSH) peaks at the initial evaluation.

The clinical parameters were compared by categorizing patients based on the difference in height (Δ, cm) from treatment cessation to menarche, and the mean Δheight was calculated and used for the subsequent analyses. Other subgroups were classified by age and the BMI at diagnosis and analyzed for height. In addition, the correlation between Δheight from treatment cessation to menarche and clinical factors such as CA, BA, height, weight, BMI, MPH, PAH, and serum LH/FSH/IGF-I levels were analyzed.

### 2.4. Statistical Analysis

Statistical analysis was performed using the Statistical Package for the Social Sciences software (ver. 23.0, IBM Corp., Armonk, NY, USA). All data are expressed as the mean ± standard deviation. To verify the normality of all data, the Shapiro–Wilk test was used. The independent t-test or Wilcoxon’s rank-sum test, depending on the data distribution, was used to compare data between subgroups by age at diagnosis and Δheight from treatment cessation to menarche. One-way analysis of variance for repeated measures followed by a post hoc Tukey’s test was performed to compare the clinical findings across the three groups stratified by BMI and the values at diagnosis, treatment cessation, and menarche. Pearson’s correlation coefficients were obtained to determine the correlation between Δheight/height SDS at menarche and clinical parameters at diagnosis, treatment discontinuation, and menarche. The factors that affected Δheight after GnRHa treatment were assessed using multivariate linear regression analysis. The dependent variable was Δheight between treatment cessation and menarche, and the independent variables were age at menarche, baseline/treatment cessation BMI SDS, and duration of treatment cessation and menarche. Statistical significance was set at *p* < 0.05. 

## 3. Results

### 3.1. Comparison of Baseline Clinical Characteristics and Laboratory Findings

CA and BA at diagnosis were 8.20 ± 0.68 and 9.80 ± 1.02 years, respectively. BA advanced 1.59 ± 0.86 years compared to CA. At the cessation of treatment, the mean BA was 11.88 ± 0.53 years, while mean BA advancement was 0.64 ± 0.57 years. At menarche, the mean CA was 12.84 ± 0.58 years and mean BA was 13.67 ± 0.86 years, while mean BA advancement was 0.83 ± 0.78 years. The mean height SDS values were 0.78 ± 0.82 at diagnosis, 0.31 ± 0.80 at treatment cessation, and 0.76 ± 0.86 at menarche, and they were statistically significant (*p* < 0.001). Weight SDS and BMI SDS values increased gradually over time, but there were no significant differences at any time point. PAH levels increased significantly throughout the diagnosis to the menarche period (*p* < 0.001). Mean MPH SDS was −0.17 ± 0.80. Mean treatment duration was 3.04 ± 0.78 years and the mean interval between treatment cessation and menarche was 1.58 ± 0.53 years (Table 1).

### 3.2. Association of Age at ICPP and EP Diagnosis with Clinical Outcomes at Menarche

Since EP patients with controversial treatment choices of GnRHa were included in this study, clinical data were compared by dividing patients into two groups based on the classic CPP diagnosis age, 8 years, to exclude errors regarding the age at which diagnosis and treatment started. A comparison of the two subgroups based on the age at diagnosis revealed that 61 girls were aged ≥8 years (EP) and 27 were aged <8 years (ICPP) (Table 2). There were no significant intergroup differences in the outcomes at menarche by age at diagnosis. Moreover, age at diagnosis was not significantly correlated with other auxological factors (data not shown).

### 3.3. Comparison of the Two Subgroups According to ΔHeight from the End of Treatment to Start of Menarche

The mean Δheight from treatment cessation to menarche was 9.79 cm ± 3.53 cm. The two subgroups were evaluated according to the Δheight from treatment cessation to menarche. Group 1 (*n* = 43) included girls with Δheight ≥ 9.79 cm, whereas Group 2 (*n* = 45) included girls with Δheight < 9.79 cm. The mean Δheight values were 12.57 cm ± 2.58 cm in group 1 and 7.13 cm ± 1.86 cm in group 2 (*p* < 0.001). The MPH SDS (*p* = 0.048) and BA advancement (BA–CA) (*p* = 0.008) at diagnosis differed significantly between the two groups. BA increased at treatment cessation and menarche in Group 1. CA, weight SDS, PAH, and BA advancement values at menarche did not differ significantly between the two groups. The BMI and BMI SDS significantly increased in group 2 at all three time points. At diagnosis and treatment cessation, the weight SDS significantly decreased in Group 1 (Table 3). 

### 3.4. Comparison of Three Subgroups According to BMI at Diagnosis

Three subgroups were stratified according to BMI at diagnosis. BMI, height SDS, and weight SDS significantly increased in the obesity group at diagnosis, whereas at menarche, height SDS remained constant across all three groups. Δheight between treatment cessation and menarche was significantly higher in the control group than in the overweight and obese groups (Table 4).

### 3.5. Factors Correlated with ΔHeight 

Δheight from treatment cessation to menarche was positively correlated with MPH and MPH SDS scores. Weight and BMI SDS at diagnosis, end of treatment, and menarche were negatively associated with Δheight. The CA at treatment cessation was negatively correlated with Δheight, whereas the CA at menarche was positively associated with Δheight. BA at diagnosis and treatment cessation was negatively correlated with Δheight, whereas BA at menarche was positively associated with Δheight. BA advancement (BA–CA) and IGF-I at diagnosis were negatively correlated with Δheight. The interval from treatment cessation to menarche positively correlated with height (Table 5). 

### 3.6. Association between BMI SDS and Height SDS

**Figure 1:**
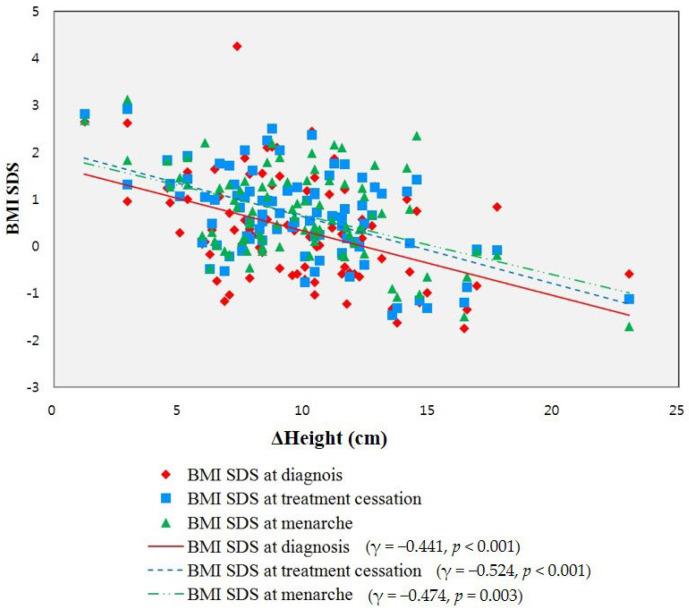
Correlation between Δheight from treatment cessation to menarche and BMI SDS at diagnosis, treatment cessation, and menarche. BMI, body mass index; SDS, standard deviation score.

The BMI SDS at treatment cessation was positively correlated with the height SDS at diagnosis and treatment cessation (Table 6). PAH was not correlated with BMI SDS at treatment cessation. BA advancement at diagnosis and at the end of treatment was positively correlated with BMI SDS at treatment cessation. The intervals and Δheight from treatment cessation to menarche were negatively correlated with the BMI SDS at treatment cessation. BMI SDSs at diagnosis and menarche were positively correlated with BMI SDS at treatment cessation. Δheight from treatment cessation to menarche was negatively associated with BMI SDS at diagnosis (*γ* = −0.441, *p* < 0.001), treatment cessation (*γ* = −0.524, *p* < 0.001), and menarche (*γ* = −0.474, *p =* 0.003). The BMI SDS at treatment cessation showed a stronger negative correlation with Δheight from treatment cessation to menarche than Δheight and BMI SDS at diagnosis or menarche (Figure 1).

### 3.7. Association between BMI SDS and ΔHeight SDS between Treatment Cessation and Menarche

Factors affecting the Δheight between treatment cessation and menarche are shown in Table 7. The four factors affecting Δheight in multiple linear regression analyses were CA at menarche, BMI SDS at diagnosis, BMI SDS at treatment cessation, and time between treatment cessation and menarche. BMI SDS at diagnosis and BMI SDS at treatment cessation were negatively associated with Δheight in ICPP and EP patients receiving GnRHa therapy (beta: −0.325, *p* < 0.001; beta: −0.365, *p* < 0.001). Δheight was positively associated with CA at menarche and the duration between treatment cessation and menarche in these patients (*p* < 0.001).

## 4. Discussion

The aim of this study was to investigate changes in growth following the completion of GnRHa treatment and those prior to menarche and determine whether this growth may be affected by the BMI in girls with ICPP and EP. Several assumptions and hypotheses have been proposed to explain the mechanism underlying the association between higher BMI and ICPP and menarche. Frisch and McArthur [35] suggested that pubertal onset (defined as menarche) requires a critical weight or fatness, but this theory remains controversial. Adipokines (e.g., leptin) has also been reported to have an important influence on puberty initiation [36,37]. Leptin, a 16-kDa protein secreted by adipocytes, has been shown to stimulate directly GnRH neurons on the hypothalamus, and modulate pulsatile activity to effects LH and FSH secretions on the pituitary gland [37]. In addition, leptin acts to stimulate enzymes instrumental in androgen synthesis in the adrenal glands, which in turn leads to higher levels of sex hormone secretion [38]. Another study reported that greater adiposity in obese peripubertal girls may be associated with the induction of peripheral adrenal androgen conversion by aromatase in fat cells [39].

The diagnosis of ICPP is based on early hypothalamic pituitary maturation with no evidence of organic brain etiologies, such as hypothalamic hamartomas and tumors [40,41]. By inhibiting pulsatile GnRH release, GnRHa therapy can preserve height potential by delaying early epiphyseal fusion [42]. 

Menarche occurs between the ages of 12 and 13 years in girls with normal development [43,44]. Peak height velocity is observed at a mean age of 11.5 years, and menarche occurs approximately a year after the growth spurt [44]. Girls generally stop growing by the end of puberty, and the increase in height after menarche is approximately 7 cm (3 in) based on the age at onset of menarche [44]. In this study, the mean CA and BA at menarche were 12.84 and 13.67 years, respectively. The BA at menarche was reportedly 12–13 years in Korean report [45]. Due to the growth spurt before menarche, studies investigating the factors influencing height between cessation of GnRH treatment and menarche may contribute to improving final adult height.

To date, several trials have analyzed the effects of GnRHa treatment using different methods. However, none of these studies have evaluated its effects on menarche. The current study differs from previous studies in that treatment outcome was analyzed by evaluating near adult height (height at menarche) and the height difference from the end of treatment to menarche. The definition of adult height used in this study differed from that used in previous studies. Most studies have evaluated treatment outcomes using the final adult height [6,46,47,48]. Some studies used the PAH at the end of therapy [5]. 

The effects of GnRHa treatment were evaluated according to the height gain (final adult height minus PAH at diagnosis) rather than the PAH at diagnosis [47,49]. Partsch et al. [47] revealed that the initial BA advancement and treatment duration were significantly associated with height gain (final adult height minus PAH at diagnosis). Additionally, height gain (final adult height minus PAH at diagnosis) was negatively correlated with CA at the start of treatment. However, it was positively correlated with BA at the start of treatment. Mul et al. [49] showed that height gain was positively associated with BA advancement at the initiation of treatment and BA at the start of treatment. Nevertheless, it was negatively associated with CA at the start of treatment. In our study, Δheight was calculated as the adult height minus the height at the end of the treatment. Girls with a higher MPH, lower weight and BMI at diagnosis and treatment cessation, and improved BA advancement at the end of treatment had a greater Δheight. CA and BA at menarche were positively correlated with Δheight. BA at diagnosis and the end of treatment, BA advancement, and IGF-I SDS score at diagnosis were negatively correlated with Δheight. A longer interval between treatment cessation and menarche was associated with a greater Δheight.

According to previous studies, girls receiving GnRHa therapy could achieve a higher final height and height gain when therapy was started at a younger age. For instance, Arrigo et al. [50] showed improved final height when the age at treatment initiation was <6 years, and Mul et al. [49] revealed a significantly higher height gain when the age at treatment initiation was <6 years instead of >8 years. In this study, age at diagnosis was not associated with height at menarche or the Δheight from the end of treatment to menarche. 

In the report on the relationship of BMI with subsequent statural growth among children born [Belarus (*n* = 16,781, born 1996 to 1997) and the United States (*n* = 1490, born 1999 to 2002)], children with higher BMI developed early puberty and slow-linearly grew up during puberty. These associations were more clearly shown in BMI measurements in late childhood [51]. In this study, even though the obese group at diagnosis had the highest height SDS, the height SDS at menarche showed no difference among three groups. As well, Δheight from the treatment cessation to menarche in the obese group showed the lowest among three groups. Some researchers showed that children with higher prepubertal BMI had a smaller pubertal height gain [52], which provides clues to our findings of slower growth between the treatment cessation to menarche among GnRHa treated early pubertal development children with higher BMI.

The correlation between BMI and growth trends after GnRHa treatment has not yet been fully elucidated. In previous studies, patients with ICPP were more likely to be overweight and obese than the general population [18,53]. However, GnRHa treatment did not cause or aggravate overweight or obesity status [18,53]. This result was also confirmed in this study, showing a negative correlation between BMI during the diagnosis to menarche period and Δheight from treatment cessation to menarche. In this study, although a gradual increase in BMI was observed, girls who presented a high BMI at diagnosis were likely to have a higher BMI at menarche. Giabicani et al. [54] showed that obesity was positively correlated with BA advancement, particularly over two years following diagnosis. In this study, BMI at the end of treatment was positively correlated with BA advancement at diagnosis and at the end of treatment. 

The current study has some limitations. The number of cases in this study was relatively small. In addition, only patients with ICPP and EP at a single hospital center were included. Therefore, the data obtained were limited. Although patients presented signs of puberty before 8 years of age, the diagnostic age of the patients ranged from 6.17 to 8.92 years, which is more limited than that reported in other studies. Despite these limitations, our results provide useful information regarding the correlation between height gain and BMI in patients with relatively early pubertal development.

## 5. Conclusions

A negative correlation was observed between the BMI and change in height from treatment cessation to menarche. This finding indicates that obesity can decrease the potential height gain in girls with early pubertal development. In conclusion, our findings from girls with early pubertal development support the role of higher BMI during the GnRHa treatment period on slower linear growth after treatment cessation. Therefore, proper BMI management prior to treatment start up to cessation of treatment is important to help improve the height gain caused by GnRHa therapy.

## Figures and Tables

**Table 1:** Clinical characteristics of 88 girls with ICPP and EP.

Variable	At Diagnosis	At Treatment Cessation	At Menarche	*p*-Value
CA (years)	8.20 ± 0.68 * (6.17–8.92)	11.25 ± 0.45 ^†^ (9.92–12.42)	12.84 ± 0.58 ^‡^ (11.25–14.33)	<0.001
BA (years)	9.80 ± 1.02 * (6.84–12.0)	11.88 ± 0.53 ^†^ (10.50–13.0)	13.67 ± 0.86 ^‡^ (11.0–15.0)	<0.001
Height (cm)	131.78 ± 5.35 *	148.0 ± 5.14 ^†^	157.8 ± 5.16 ^‡^	<0.001
Weight (kg)	30.85 ± 6.25 *	45.04 ± 8.10 ^†^	53.31 ± 8.11 ^‡^	<0.001
BMI (kg/m^2^)	17.68 ± 0.89 *	20.48 ± 2.98 ^†^	21.39 ± 2.92 ^†^	<0.001
Height SDS	0.78 ± 0.82 *	0.31 ± 0.80 ^†^	0.76 ± 0.86 *	<0.001
Weight SDS	0.61 ± 0.94	0.66 ± 0.88	0.84 ± 0.84	0.142
BMI SDS	0.35 ± 1.10	0.65 ± 0.96	0.68 ± 0.94	0.056
PAH (cm)	158.84 ± 5.49 *	161.42 ± 5.28 ^†^	163.25 ± 4.81 ^†^	<0.001
BA–CA (years)	1.59 ± 0.86 *	0.64 ± 0.57 ^†^	0.83 ± 0.78 ^†^	<0.001
MPH (cm)	159.91 ± 3.91			
MPH SDS	−0.17 ± 0.80			
Duration (years)	3.04 ± 0.78 (1.59–4.66) between diagnosis and treatment cessation		1.58 ± 0.53 (0.25–3.33) between treatmenet cessation and menarche	

Values are presented as mean ± standard deviation and ranges (–). CA, chronological age; BA, bone age; BMI, body mass index; SDS, standard deviation score; PAH, predicted adult height; MPH, mid-parental height. *^, †, ‡^ Identical subscript letters indicate a non-significant difference between groups based on Tukey’s multiple comparison test.

**Table 2:** Comparison of outcomes at menarche classified by age at the diagnosis.

Variables	Age < 8 Years (ICPP)	Age ≥ 8 Years (EP)	*p*-Value
Number	27	61	
CA at diagnosis (years)	7.36 ± 0.47 (6.17–7.92)	8.58 ± 0.32 (8.00–8.92)	<0.001
CA at menarche (years)	12.80 ± 0.58 (11.25–13.90)	12.86 ± 0.58 (11.42–14.33)	0.653
BA–CA at menarche (years)	0.78 ± 0.69	0.86 ± 0.83	0.642
Height at menarche SDS	0.79 ± 1.02	0.75 ± 0.78	0.858
PAH at menarche (cm)	163.97 ± 5.10	162.94 ± 4.68	0.373
Δheight (cm)	9.72 ± 3.47	9.82 ± 3.58	0.901
Duration between treatment cessation and menarche (years)	1.53 ± 0.49	1.61 ± 0.55	0.540

Values are presented as mean ± standard deviation and ranges (–). Δheight was calculated as the difference between the near adult height and the height at the end of the treatment. ICPP, idiopathic central precocious puberty; EP, central early puberty; CA, chronological age; BA, bone age; PAH, predicted adult height; SDS, standard deviation score.

**Table 3:** Auxological outcomes of the two subgroups according to Δheight from treatment cessation to menarche.

Variables	At Diagnosis			At Treatment Cessation			At Menarche		
	Group 1 (≥9.79 cm)	Group 2 (<9.79 cm)	*p*-Value	Group 1 (≥9.79 cm)	Group 2 (<9.79 cm)	*p*-Value	Group 1 (≥9.79 cm)	Group 2 (<9.79 cm)	*p*-Value
Number	43	45		43	45		43	45	
Δheight (cm)							12.57 ± 2.58	7.13 ± 1.86	<0.001
CA (years)	8.26 ± 0.71	8.15 ± 0.65	0.474	11.11 ± 0.45	11.39 ± 0.40	0.004	12.95 ± 0.55	12.73 ± 0.59	0.069
BA (years)	9.61 ± 1.10	9.98 ± 0.96	0.084	11.63 ± 0.54	12.12 ± 0.40	<0.001	13.90 ± 0.83	13.44 ± 0.85	0.012
BMI (kg/m^2^)	16.76 ± 2.38	18.57 ± 3.08	0.003	19.27 ± 2.70	21.63 ± 2.80	<0.001	20.71 ± 2.84	22.05 ± 2.87	0.031
Height (cm)	131.39 ± 5.58	132.16 ± 5.15	0.501	146.70 ± 5.47	149.26 ± 4.51	0.019	159.27 ± 5.54	156.39 ± 4.38	0.008
Height SDS	0.67 ± 0.84	0.90 ± 0.79	0.174	0.21 ± 0.83	0.41 ± 0.77	0.260	0.89 ± 0.91	0.64 ± 0.79	0.178
Weight SDS	0.27 ± 0.88	0.95 ± 0.89	0.001	0.34 ± 0.84	0.97 ± 0.82	0.001	0.77 ± 0.88	0.95 ± 0.81	0.315
BMI SDS	−0.06 ± 0.97	0.73 ± 1.09	0.001	0.28 ± 0.96	1.01 ± 0.83	<0.001	0.45 ± 0.98	0.89 ± 0.86	0.026
PAH (cm)	159.39 ± 5.88	158.31 ± 5.09	0.362	161.07 ± 5.64	161.76 ± 4.96	0.550	163.99 ± 5.18	162.55 ± 4.36	0.165
BA–CA (years)	1.35 ± 0.82	1.83 ± 0.84	0.008	0.54 ± 0.57	0.73 ± 0.57	0.117	0.95 ± 0.76	0.72 ± 0.80	0.162
MPH SDS	0.00 ± 0.67	−0.34 ± 0.89	0.048						

Values are presented as mean ± standard deviation. CA, chronological age; BA, bone age; BMI, body mass index; PAH, predicted adult height; MPH, mid-parental height; SDS, standard deviation score.

**Table 4:** Auxological outcomes of the three subgroups according to BMI at diagnosis.

Variable		Control	Overweight	Obesity	*p*-Value
Number		65	13	10	
At diagnosis	CA (years)	8.21 ± 0.68	8.25 ± 0.66	8.09 ± 0.73	0.843
	BA (years)	9.79 ± 0.96	9.94 ± 1.02	9.67 ± 1.34	0.810
	BMI (kg/m^2^)	16.35 ± 1.37 *	19.91 ± 0.53 ^†^	23.44 ± 3.18 ^‡^	<0.001
	Height SDS	0.70 ± 0.85 *	0.70 ± 0.41 *	1.45 ± 0.79 ^†^	0.025
	Weight SDS	0.23 ± 0.71 *	1.24 ± 0.26 ^†^	2.29 ± 0.44 ^‡^	<0.001
	MPH (cm)	160.03 ± 4.03	158.62 ± 2.99	160.84 ± 4.08	0.363
At menarche	CA (years)	12.84 ± 0.55	13.02 ± 0.64	12.56 ± 0.65	0.161
	BA (years)	13.63 ± 0.77	13.96 ± 0.99	13.53 ± 1.26	0.393
	BMI (kg/m^2^)	20.35 ± 2.11 *	23.63 ± 2.68 ^†^	25.28 ± 3.01 ^†^	<0.001
	Height SDS	0.70 ± 0.81	0.70 ± 0.68	1.19 ± 0.20	0.232
	Weight SDS	0.62 ± 0.66	1.24 ± 0.87	1.96 ± 0.88	<0.001
Δheight (cm) between treatment cessation and menarche		10.21 ± 3.55 *	9.66 ± 0.98 *	7.19 ± 3.17 ^†^	0.039

Values are presented as mean ± standard deviation. Δheight was calculated as the difference between the near adult height and the height at the end of treatment. Near-adult height is the height at menarche. CA, chronological age; BA, bone age; BMI, body mass index; SDS, standard deviation score; PAH, predicted adult height; MPH, mid-parental height. *^, †, ‡^ Identical subscript letters indicate a non-significant difference between groups based on the Tukey multiple comparison test.

**Table 5:** Correlation between Δheight from treatment cessation to menarche and different parameters.

Variables	At Diagnosis		At Treatment Cessation		At Menarche	
	*p*-Value	*γ*	*p*-Value	*γ*	*p*-Value	*γ*
MPH	0.007	0.284				
MPH SDS	0.016	0.256				
PAH	0.353	0.100	0.170	−0.148	0.063	0.199
Height SDS	0.011	−0.271	0.192	−0.140	0.256	0.122
Weight SDS	<0.001	−0.522	<0.001	−0.474	0.002	−0.328
BMI SDS	<0.001	−0.441	<0.001	−0.524	0.003	−0.474
CA	0.645	0.050	<0.001	−0.383	0.002	0.326
BA	0.014	−0.261	<0.001	−0.586	0.009	0.278
BA–CA	0.001	−0.348	0.059	−0.202	0.546	0.065
LH base	0.576	−0.060				
LH peak	0.050	−0.210				
Peak LH/FSH ratio	0.344	−0.102				
IGF-I SDS	0.006	−0.291				
Interval between treatment cessation and menarche					<0.001	0.680

Values are presented as *γ* values (*p* values). MPH, mid-parental height; PAH, predicted adult height; BMI, body mass index; CA, chronological age; BA, bone age; LH, luteinizing hormone; FSH, follicle-stimulating hormone; IGF-I, insulin-like growth factor I; SDS, standard deviation score.

**Table 6:** Correlation between BMI SDS at treatment cessation and different parameters.

Variables	At Diagnosis		At Treatment Cessation		At Menarche	
	*p*-Value	*γ*	*p*-Value	*γ*	*p*-Value	*γ*
BMI SDS	<0.001	0.783			<0.001	0.822
Height SDS	0.002	0.323	0.024	0.241	0.274	0.118
PAH	0.934	−0.009	0.147	0.156	0.728	−0.038
BA–CA	0.026	0.238	0.001	0.344	0.204	0.137
Duration between treatment cessation and menarche					<0.001	−0.366
Δheight from treatment cessation to menarche					<0.001	−0.524

Values are presented as *γ* values (*p* values). BMI, body mass index; SDS, standard deviation score; PAH, predicted adult height; BA, bone age; CA, chronological age. Δheight was calculated as the difference between the near adult height and the height at the end of treatment.

**Table 7:** Multiple stepwise regression analysis for Δheight, clinical variables and BMI.

	Model 1 ^a^				Model 2 ^b^			
	B	SE	β	*p*-Value	B	SE	β	*p*-Value
CA at menarche	−1.623	0.573	−0.267	0.006	−2.028	0.574	−0.334	0.001
BMI SDS at diagnosis	−1.041	0.227	−0.325	<0.001	-	-	-	-
BMI SDS at treatment cessation	-	-	-	-	−1.342	0.274	−0.365	<0.001
Duration between treatment cessation and menarche	5.295	0.632	0.796	<0.001	5.143	0.628	0.773	<0.001

^a^ Model 1: Dependent variable, Δheight between treatment cessation and menarche; independent variables, CA at menarche, BMI SDS at diagnosis, and duration between treatment cessation and menarche; r^2^, 0.597. ^b^ Model 2: Dependent variable, Δheight between treatment cessation and menarche; Independent variables, CA at menarche, BMI SDS at treatment cessation, and duration between treatment cessation and menarche; r^2^, 0.608.

## Data Availability

The data used in this study are available from the corresponding authors upon request.
